# Coexisting Behaviors of Asymmetric Attractors in Hyperbolic-Type Memristor based Hopfield Neural Network

**DOI:** 10.3389/fncom.2017.00081

**Published:** 2017-08-23

**Authors:** Bocheng Bao, Hui Qian, Quan Xu, Mo Chen, Jiang Wang, Yajuan Yu

**Affiliations:** School of Information Science and Engineering, Changzhou University Changzhou, China

**Keywords:** hyperbolic-type memristor, Hopfield neural network (HNN), asymmetric attractors, coexisting behaviors, hardware experiment

## Abstract

A new hyperbolic-type memristor emulator is presented and its frequency-dependent pinched hysteresis loops are analyzed by numerical simulations and confirmed by hardware experiments. Based on the emulator, a novel hyperbolic-type memristor based 3-neuron Hopfield neural network (HNN) is proposed, which is achieved through substituting one coupling-connection weight with a memristive synaptic weight. It is numerically shown that the memristive HNN has a dynamical transition from chaotic, to periodic, and further to stable point behaviors with the variations of the memristor inner parameter, implying the stabilization effect of the hyperbolic-type memristor on the chaotic HNN. Of particular interest, it should be highly stressed that for different memristor inner parameters, different coexisting behaviors of asymmetric attractors are emerged under different initial conditions, leading to the existence of multistable oscillation states in the memristive HNN. Furthermore, by using commercial discrete components, a nonlinear circuit is designed and PSPICE circuit simulations and hardware experiments are performed. The results simulated and captured from the realization circuit are consistent with numerical simulations, which well verify the facticity of coexisting asymmetric attractors' behaviors.

## Introduction

The neurons regarded as the fundamental component unit of brain can generate intricate dynamical behaviors (Korn and Faure, 2003; Ma and Tang, 2017) and the constructing Hopfield neural network (HNN) (Hopfield, 1984) is a significant model in artificial neurology. When a nonlinear function is taken for a neuron activation function, the HNN belongs to a nonlinear dynamical system (Hopfield, 1984), resulting in the generations of chaos, hyperchaos, period, and/or quasi-period. In the past few years, numerous results with respect to the complex dynamical behaviors of the HNN have been reported generally (Bersini and Sener, 2002; Li et al., 2005; Yang and Huang, 2006; Yuan et al., 2009; Zheng et al., 2010; Danca and Kuznetsov, 2017). As a whole, it is necessary and pregnant to further investigate complex dynamics in the neural network (Korn and Faure, 2003), particularly the HNN with a memristive synaptic weight (Li et al., 2014).

With the appearance of memristor (Strukov et al., 2008), it gradually generates far-reaching influence in the field of circuit fundamental theory. Since memristor is a nonlinear two-terminal element (Chua, 2012), a large number of research achievements with respect to memristive chaotic circuits have been reached by introducing different memristors into some classical application circuits (Bao et al., 2014, 2015, 2016a,b; Chen et al., 2015; Kengne et al., 2015; Li et al., 2015; Wu et al., 2016; Xu et al., 2016; Zhou et al., 2017). Similarly, memristor can be also applied to the artificial neural network because the characteristic of neuron synapse is similar to the feature of memristor (Chua et al., 2012; Kim et al., 2012; Prezioso et al., 2015; Wang Z. R. et al., 2016), which has been drawn broad researchers' more and more attention in recent years (Wang et al., 2011; Adhikari et al., 2012; Li et al., 2014; Duan et al., 2015; Pham et al., 2016; Wang L. D. et al., 2016; Yang et al., 2017). These researches in physiology and anatomy are more effective to help us understand the dynamical properties of a large network (Korn and Faure, 2003). Hence, it is extremely significant and indispensable to investigate chaotic dynamics of memristive neural networks for studying brain functions as well as artificial neural networks (Guckenheimer and Oliva, 2002; Li et al., 2005; Wang et al., 2017).

By introducing a memristive synaptic weight (Li et al., 2014; Pham et al., 2016) to substitute a resistive synaptic weight, two kinds of memristive neural networks are presented, from which complex dynamical behaviors of self-exited quasi-periodic limit cycle, chaotic attractor, and hyperchaotic attractor as well as hidden hyperchaotic attractor are exhibited by numerical simulations. The circuit realization of the memristive neural network using discrete electronic components is developed in Pham et al. (2016), only PSPICE circuit simulations are performed to confine numerical simulations, however, without hardware experimental fabrications and measurements. In this paper, we present a hyperbolic-type memristor based 3-neuron Hopfield neural network (HNN) on the basis of the reference (Zheng et al., 2010), which can show the stabilization effect of the hyperbolic-type memristor on the chaotic HNN, resulting in a dynamical transition from chaotic, to periodic, and further to stable point behaviors with the variations of the memristor inner parameter. Especially, for different memristor inner parameters, different coexisting behaviors of asymmetric attractors are emerged under different initial conditions, which are availably validated by PSPICE circuit simulations and hardware experiments. It should be highlighted that the new finding of coexisting asymmetric attractors' behaviors in the memristive HNN and the correspondingly hardware experimental verifications have not been previously reported.

The rest of the paper is organized as follows. In Section Hyperbolic-type Memristor Emulator, a hyperbolic-type memristor emulator is proposed and then its frequency-dependent pinched hysteresis loops are exhibited by numerical simulations and hardware experiments. In Section Hyperbolic-type Memristor based HNN, a novel hyperbolic-type memristor based 3-neuron Hopfield neural network (HNN) is constructed and the stability analyses of its equilibrium points are conducted. In Section Coexisting Behaviors of Asymmetric Attractors, coexisting behaviors of asymmetric attractors are investigated by bifurcation diagrams, Lyapunov exponent spectra, and phase portraits, which exhibits the existence of multistable oscillation states in the memristive HNN. In Section PSPICE Circuit Simulations and Hardware Experiments, an implementation circuit is designed, upon which PSPICE circuit simulations and hardware experiments are performed to verify the coexisting behaviors of asymmetric attractors in the hyperbolic-type memristor based HNN. The conclusions are summarized in Section Conclusion.

## Hyperbolic-type memristor emulator

A neuron activation function is a monotone differentiable function which is bounded above and below. Therefore, a hyperbolic tangent function is usually utilized as the neuron activation function. For this reason, a new hyperbolic-type memristor emulator is proposed and its constitutive relation is modeled as

(1) $$\begin{array}{l} \;i=W(v_{0})v=[a-b\tanh(v_{0})]v\\ \tau dv_{0}/d\text{t}=f(v_{0},v)=-v_{0}-v \end{array}$$

where *v* and *i* represent the voltage and current at the input port of the memristor emulator, respectively, *v*_0_ is the inner state variable, *a* and *b* are two inner positive constants of the memristor emulator, and τ is integral time constant. The nonlinear memductance function *W*(*v*_0_) can be expressed as

(2) $$W(v_{0})=a-b\tanh(v_{0})$$

which implies that the memristor is passive and voltage-controlled. Thus, the proposed emulator can be defined as a non-ideal hyperbolic-type memristor emulator.

**Figure 1:**
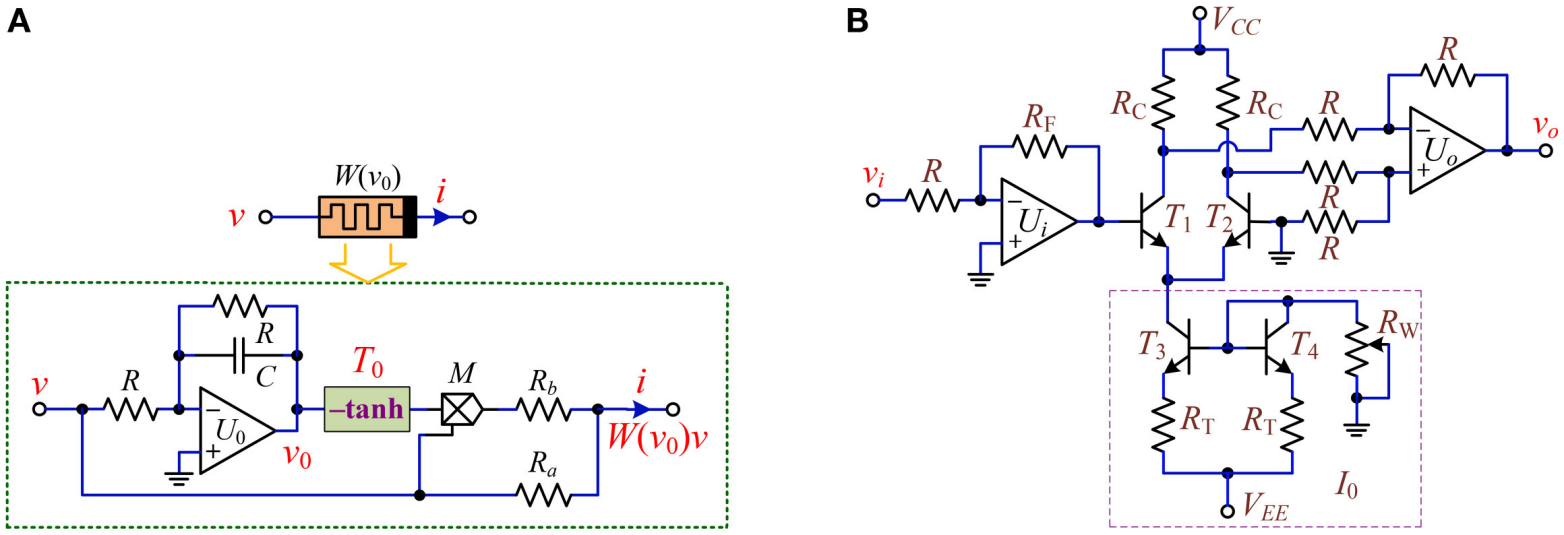
Circuit realization of the hyperbolic-type memristor emulator. **(A)** Circuit realization scheme; **(B)** the inverting hyperbolic tangent function unit circuit.

With off-the-shelf discrete components, the circuit realization scheme of the hyperbolic-type memristor emulator described by Equation (1) is designed, as shown in Figure 1. The realization circuit shown in Figure 1A consists of an integrator *U*_0_ connected two resistors *R* and a capacitor *C*, an inverting hyperbolic tangent function circuit unit *T*_0_ marked by –tanh with solid box, an analog multiplier *M* and two resistors *R*_*a*_ and *R*_*b*_. It is pointed that different from the ideal voltage-controlled memristor reported in Bao et al. (2014, 2015, 2016a,b) and Li et al. (2015), a resistor *R* is newly added to the integrating capacitor *C* in parallel to avoid DC voltage integral drift. The hyperbolic tangent function circuit unit shown in Figure 1B is implemented by a dual-transistor pair of *T*_1_ and *T*_2_, a module of current source *I*_0_, and two operational amplifier circuits for controlling gains (Duan and Liao, 2007).

Three operational amplifiers TL082CP and one analog multiplier AD633JNZ with ±15 V DC voltage sources, four crucial bipolar transistors MPS2222, fifteen precision potentiometers, and one monolithic ceramic capacitor are utilized. The linear element parameters in Figure 1 are employed as listed in Table 1. With the element parameters, the output of the hyperbolic tangent function circuit unit is thus obtained as Duan and Liao (2007).

(3) $$v_{o}=-\tanh(v_{i})$$

where *v*_*i*_ and *v*_*o*_ stand for the input voltage and output voltage of the circuit in Figure 1B, respectively.

**Table 1:** Circuit parameters of the memristor emulator for simulations and experiments.

**Parameter**	**Signification**	**Value**
*R*	Resistance	10 kΩ
*C*	Capacitance	100 nF
*R_*a*_*	Resistance	10 kΩ
*R_*b*_*	Resistance	2 kΩ
*g*	Multiplier gain	0.1
*R*_F_	Resistance	520 Ω
*R*_C_	Resistance	1 kΩ
*R*_T_	Resistance	2 kΩ
*R*_W_	Resistance	9.8 kΩ

Applying Kirchhoff's circuit laws and the constitutive relations to the circuit in Figure 1A, the mathematical model of the hyperbolic-type memristor emulator can be established as

(4) $$\begin{array}{l} i=W(v_{0})v=\left[\frac{1}{R_{a}}-\frac{g}{R_{b}}\tanh(v_{0})\right]v\\ \;\frac{dv_{0}}{dt}=f(v_{0},v)=-\frac{1}{RC}(v_{0}+v) \end{array}$$

where *g* is the gain of the multiplier *M*. Introducing the hyperbolic-type memristor emulator into a dynamical system and scaling the circuit parameters in a dimensionless form, there yields

(5) $$\tau =t/RC,a=R/R_{a},b=gR/R_{b}$$

The circuit parameters of the hyperbolic-type memristor emulator are listed in Table 1. Thus, the normalized parameters of the nonlinear memductance function *W*(*v*_0_) are calculated as *a* = 1 and *b* = 0.5.

**Figure 2:**
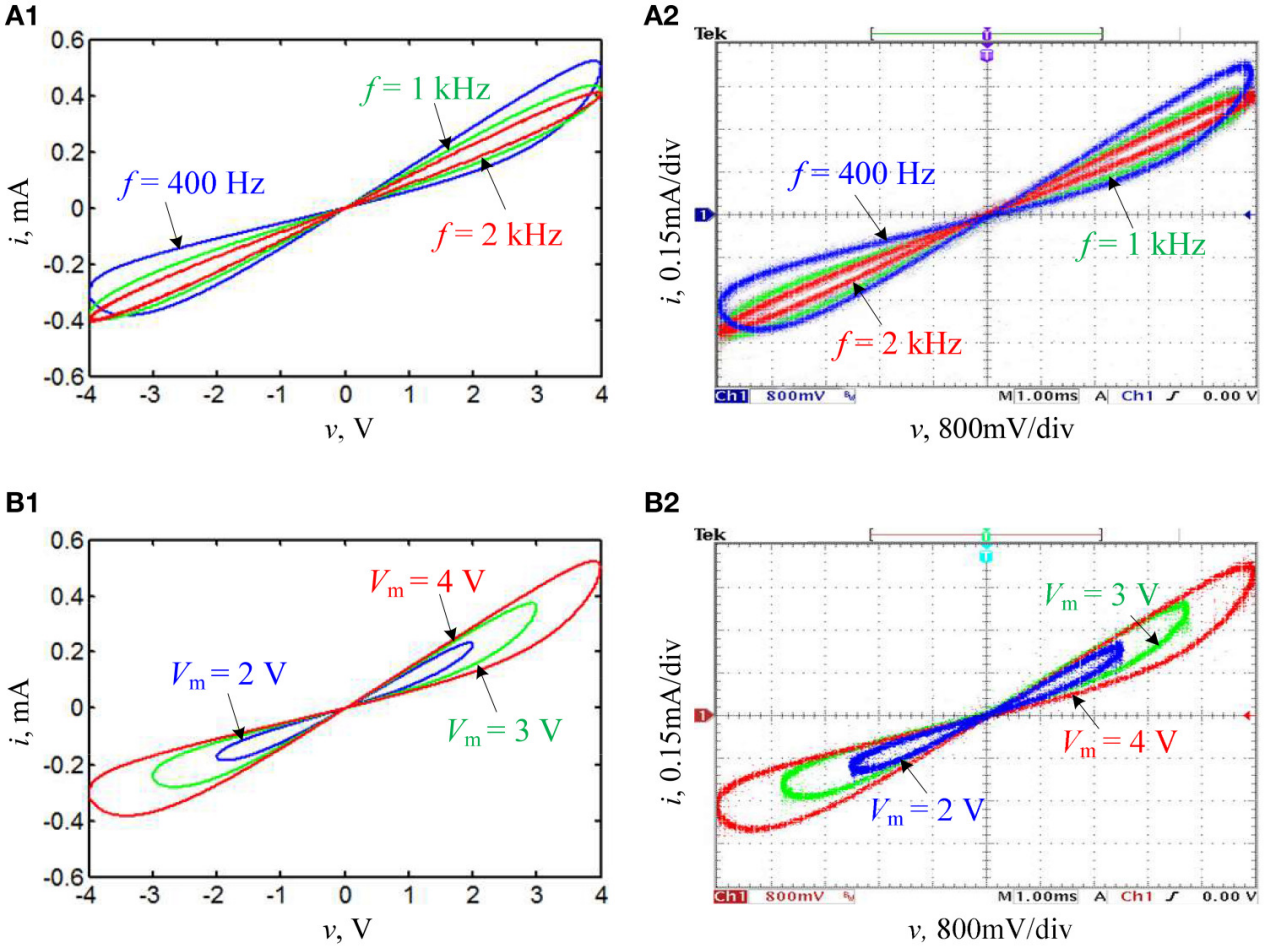
Numerically simulated and experimentally measured pinched hysteresis loops of the hyperbolic-type memristor emulator, where **(A1,B1)** are numerical simulations and **(A2,B2)** are experimental measurements. **(A)** *V*_m_ = 4 V with different stimulus frequencies; **(B)** *f* = 400 Hz with different stimulus amplitudes.

For the sake of verifying the frequency-dependent pinched hysteresis loops of the hyperbolic-type memristor emulator, the circuit parameters given in Table 1 are selected and a sinusoidal voltage source *v* = *V*_m_sin(2π*ft*) is considered, where *V*_m_ and *f* are the stimulus amplitude and frequency, respectively. When *V*_m_ = 4 V is held and *f* is set to 400 Hz, to 1 kHz, and to 2 kHz, respectively, the *v* – *i* curves are plotted in Figure 2A1, which exhibits that the hysteresis loop is pinched at the origin and shrinks into a single-valued function at infinite frequency, and its lobe area decreases with the increase of the frequency. While when *f* = 400 Hz is maintained and *V*_m_ is set to 2 V, to 3 V, and to 4 V, respectively, the *v* – *i* curves are plotted in Figure 2B1, which explains that the pinched hysteresis loop is regardless of the stimulus amplitude. The numerical simulations in Figure 2 demonstrate that the hyperbolic-type memristor emulator can behave three fingerprints for distinguishing memristors (Adhikari et al., 2013).

With the circuit scheme given in Figure 1, a hardware level on breadboards is made to verify the numerical simulations. In hardware experiments, the linear element parameters and the stimulus parameters of the sinusoidal voltage source are the same as those used in numerical simulations. In addition, a function signal generator is used as the applied sinusoidal voltage source and a 4 channel digital oscilloscope is utilized to capture the experimental phase portraits. Consequently, the pinched hysteresis loops for various stimulus frequencies and stimulus amplitudes are captured as shown in Figures 2A2,B2, respectively, which illustrates that the measurement results from the hardware circuit are well consistent to those revealed by numerical simulations.

## Hyperbolic-type memristor based HNN

### Mathematical modeling

Hopfield neural network (HNN) can be featured by a set of circuit state equations corresponded to *n* neurons (Bersini and Sener, 2002). For the *i*-th neuron, the circuit state equation is expressed as

(6) $$C_{i}\frac{dx_{i}}{dt}=-\frac{x_{i}}{R_{i}}+\sum_{j\;=\;1}^{n}w_{ij}\tanh(x_{j})+I_{i}$$

where *x*_*i*_ is a state variable standing for the voltage across the capacitor *C*_*i*_, *R*_*i*_ is a resistor representing the membrane resistance between the inside and outside of the neuron, *I*_*i*_ is a input bias current, tanh(*x*_*j*_) is a neuron activation function indicating the voltage input from the *j*-th neuron, and ***W*** = (*w*_*ij*_) is an *n* × *n* synaptic weight matrix illustrating the strength of connections between the *i*-th and *j*-th neurons.

In the realization circuit of HNN, the synaptic weight *w*_*ij*_ is generally constructed by a resistor connecting the *i*-th and *j*-th neurons. When the connection resistor is replaced by the above hyperbolic-type memristor characterized by Equation (1), a memristor based HNN can be easily proposed.

**Figure 3:**
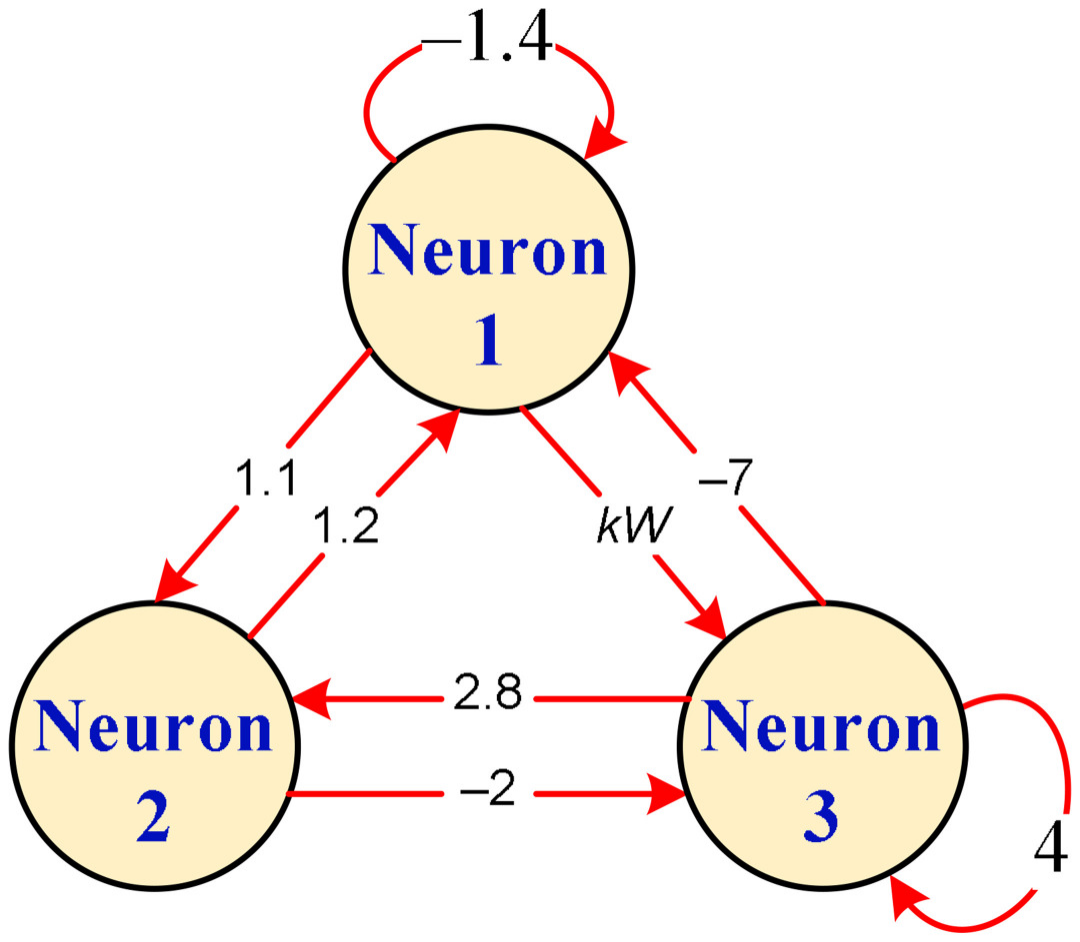
Connection topology for hyperbolic-type memristor based HNN.

A hyperbolic-type memristor based HNN with 3 neurons is considered in our next work. The connection topology for the hyperbolic-type memristor based HNN is displayed in Figure 3. Corresponding to this connection topology, the connection matrix considered is of the following form

(7) $$W=\left[\begin{array}{ccc} w_{11} & w_{21} & w_{31}\\ w_{12} & w_{22} & w_{32}\\ w_{13} & w_{23} & w_{33} \end{array}\right]=\left[\begin{array}{ccc} -1.4 & 1.2 & -7\\ 1.1 & 0 & 2.8\\ kW & -2 & 4 \end{array}\right]$$

Define *n* = 3, *C*_*i*_ = 1, *R*_*i*_ = 1, *I*_*i*_ = 0, and *RC* = 1. The autonomous ordinary differential equations describing the proposed memristive network can be derived in a dimensionless form as

(8) $$\begin{array}{l} {\dot{x}}_{1}=-x_{1}-1.4\tanh(x_{1})+1.2\tanh(x_{2})-7\tanh(x_{3})\\ {\dot{x}}_{2}=-x_{2}+1.1\tanh(x_{1})+2.8\tanh(x_{3})\\ {\dot{x}}_{3}=-x_{3}+kW\tanh(x_{1})-2\tanh(x_{2})+4\tanh(x_{3})\\ {\dot{x}}_{4}=-x_{4}+\tanh(x_{1}) \end{array}$$

where *W* = *a* – *b*tanh(*x*_4_) stands for the synaptic weight *w*_13_ connecting the first and third neurons and *k* is a positive constant representing the coupling strength of the hyperbolic-type memristor. Thus, the hyperbolic-type memristor based HNN can be modeled by Equation (8), which is a four-dimensional autonomous nonlinear dynamical system.

With respect to Equation (8), the parameters of *a, b, k* which are related to the hyperbolic-type memristor *W* are positive. It can be proved that the orbits of Equation (8) including periodic and chaotic orbits are confined in a bounded region.

Referring to the approach in Nik et al. (2015), Zahedi and Nik (2015), Chen et al. (2017), and Singh and Roy (2017), a Lyapunov function is introduced as

(9) $$V(x_{1},x_{2},x_{3},x_{4})=\frac{1}{2}x_{1}^{2}+\frac{1}{2}x_{2}^{2}+\frac{1}{2}x_{3}^{2}+\frac{1}{2}x_{4}^{2}$$

The time derivative of Equation (9) is yield as

(10) $$\begin{array}{l} \dot{V}(x_{1},x_{2},x_{3},x_{4})=x_{1}{\dot{x}}_{1}+x_{2}{\dot{x}}_{2}+x_{3}{\dot{x}}_{3}+x_{4}{\dot{x}}_{4}\\ \;=-x_{1}^{2}-x_{2}^{2}-x_{3}^{2}-x_{4}^{2}+(-1.4x_{1}+1.1x_{2}\\ \;+\;kWx_{3}+x_{4})\tanh(x_{1})\\ \;+(1.2x_{1}-2x_{3})\tanh(x_{2})+(-7x_{1}+2.8x_{2}\\ \;+\;4x_{3})\tanh(x_{3}) \end{array}$$

For convenience, denote

(11) $$\begin{array}{l} \;v(x_{1},x_{2},x_{3,}x_{4})=(-1.4x_{1}+1.1x_{2}+kWx_{3}+x_{4})\tanh(x_{1})\\ \;+\;(1.2x_{1}-2x_{3})\tanh(x_{2})+(-7x_{1}+2.8x_{2}\\ \;+\;4x_{3})\tanh(x_{3}) \end{array}$$

Equation (10) can be then rewritten as

(12) $$\dot{V}(x_{1},x_{2},x_{3},x_{4})=-2V(x_{1},x_{2},x_{3},x_{4})+v(x_{1},x_{2},x_{3},x_{4})$$

For*x*_4_ ∈ *R*,−1 < tanh(*x*_4_) < 1. Therefore,

(13) $$a-b<W=a-b\tanh(x_{4})<a+b$$

and

(14) |W|≤M=max{|a−b|,|a+b|}

For any *x*_*i*_ (*i* = 1, 2, 3, 4), one has|tanh(*x*_*i*_)| < 1. Equation (11) can be thereby simplified as

(15) $$\begin{array}{l} v(x_{1},x_{2},x_{3,}x_{4})\leq \left|\left(-1.4x_{1}+1.1x_{2}+kWx_{3}+x_{4})\tanh(x_{1}\right)\right|\\ \;+\left|\left(1.2x_{1}-2x_{3})\tanh(x_{2}\right)\right|\\ \;+\;\left|\left(-7x_{1}+2.8x_{2}+4x_{3})\tanh(x_{3}\right)\right|\\ \;<\;\left|-1.4x_{1}+1.1x_{2}+kWx_{3}+x_{4}\right|+\left|1.2x_{1}-2x_{3}\right|\\ \;+\;\left|-7x_{1}+2.8x_{2}+4x_{3}\right|\\ \;\leq 9.6\left|x_{1}\right|+3.9\left|x_{2}\right|+(kM+6)\left|x_{3}\right|+\left|x_{4}\right| \end{array}$$

Let *D*_0_ > 0 be the sufficiently large region. For all (*x*_1_, *x*_2_, *x*_3_, *x*_4_) satisfying *V*(*x*_1_, *x*_2_, *x*_3_, *x*_4_) = *D* with *D* > *D*_0_, there exists the following condition

(16) $$\begin{array}{l} v(x_{1},x_{2},x_{3,}x_{4})<9.6\left|x_{1}\right|+3.9\left|x_{2}\right|+(kM+6)\left|x_{3}\right|+\left|x_{4}\right|\\ \;<x_{1}^{2}+x_{2}^{2}+x_{3}^{2}+x_{4}^{2}=2V(x_{1},x_{2},x_{3},x_{4}) \end{array}$$

where (*kM* + 6) is a positive constant.

Consequently, on the surface

(17) $$\left\{(x_{1},x_{2},x_{3,}x_{4})|V(x_{1},x_{2},x_{3,}x_{4})=D\right\}$$

with *D* > *D*_0_, there yields

(18) $$\dot{V}(x_{1},x_{2},x_{3,}x_{4})=-2V(x_{1},x_{2},x_{3},x_{4})+v(x_{1},x_{2},x_{3,}x_{4})<0$$

which implies that the set

(19) $$\left\{(x_{1},x_{2},x_{3,}x_{4})|V(x_{1},x_{2},x_{3,}x_{4})\leq D\right\}$$

is a confined region of all solutions of Equation (8), i.e., the memristive system Equation (8) is bounded.

### Equilibrium point and stability analysis

Since the memristive system Equation (8) is invariant under the transformation (*x*_1_, *x*_2_, *x*_3_, *x*_4_, *b*) to (–*x*_1_, –*x*_2_, –*x*_3_, –*x*_4_, –*b*), the memristive system Equation (8) is symmetric about the parameter *b*.

By setting the left-hand side of Equation (8) to zero, the equilibrium points of the hyperbolic-type memristor based Hopfield neural network can be numerically solved and determined as a zero equilibrium point *P*_0_ = (0, 0, 0, 0) and two nonzero equilibrium points *P*_1_ = (δ_1_, δ_2_, δ_3_, δ_4_) and *P*_2_ = (η_1_, η_2_, η_3_, η_4_), where the nonzero equilibrium points *P*_1_ and *P*_2_ can be calculated by solving the following equations

(20a) $$\{\begin{array}{ll} \delta_{1} & =y_{1}\\ \delta_{2} & =z_{1}\\ \delta_{3} & =\text{atanh}(\frac{1}{2.8}z_{1}-\frac{1.1}{2.8}\tanh y_{1})\\ \delta_{4} & =\tanh y_{1} \end{array}$$

and

(20b) $$\{\begin{array}{ll} \eta_{1} & =y_{2}\\ \eta_{2} & =z_{2}\\ \eta_{3} & =\text{atanh}(\frac{1}{2.8}z_{2}-\frac{1.1}{2.8}\tanh y_{2})\\ \eta_{4} & =\tanh y_{2} \end{array}$$

where the values of *y*_1_, *z*_1_ and *y*_2_, *z*_2_ are the two intersection points of the two following function curves

(21a) $$\begin{array}{l} h_{1}(y,z)=\;-y-1.4\tanh(y)+1.2\tanh(z)\\ \;-\;7\tanh\{\text{atanh}[\frac{5}{14}z-\frac{11}{28}\tanh(y)]\} \end{array}$$

(21b) $$\begin{array}{l} h_{2}(y,z)=-\text{atanh}[\frac{5}{14}z-\frac{11}{28}\tanh(y)]\\ \;+\;0.8\{a-b\tanh[\tanh(y)]\}\tanh(y)\\ \;-2\tanh(z)+4\tanh\{\text{atanh}[\frac{5}{14}z-\frac{11}{28}\tanh(y)]\} \end{array}$$

**Figure 4:**
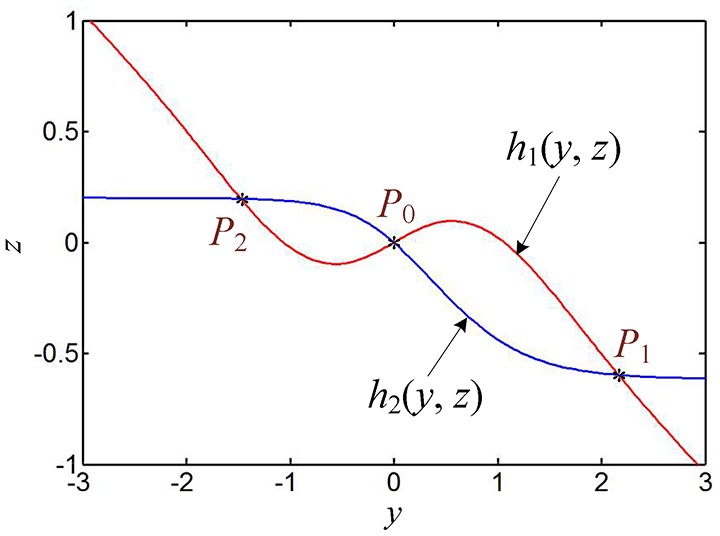
Two function curves and their intersection points, where *a* = 1 and *b* = 0.26.

Therefore, the values of *y*_1_, *z*_1_ and *y*_2_, *z*_2_ can be determined through graphic analytic method. Taking *k* = 0.8, *a* = 1, and *b* = 0.26 as an example, the two function curves given in Equation (21) can be plotted in Figure 4, from which the solutions are gotten as *y*_1_ = 2.1661, *z*_1_ = −0.5971, *y*_2_ = −1.4755, and *z*_2_ = 0.1974. Correspondingly, the two nonzero equilibrium points *P*_1_ and *P*_2_ can be easily obtained from Equation (20a) and (20b).

The Jacobian matrix at the equilibrium point $P\;=\;\left({\overset{-}{x}}_{1},{\overset{-}{x}}_{2},{\overset{-}{x}}_{3},{\overset{-}{x}}_{4}\right)$ is derived from Equation (8) as

(22) $$J=\left[\begin{array}{cccc} -1-1.4h_{1} & 1.2h_{2} & -7h_{3} & 0\\ 1.1h_{1} & -1 & 2.8h_{3} & 0\\ k\bar{W}h_{1} & -2h_{2} & -1+4h_{3} & -kb\text{tanh}({\bar{x}}_{1})h_{4}\\ h_{1} & 0 & 0 & -1 \end{array}\right]$$

where $h_{i}=\text{sec}{\text{h}}^{2}\left({\overset{-}{x}}_{i}\right)\left(i=1,2,3,4\right)$ and$\overset{-}{W}=a-b\tanh\left({\overset{-}{x}}_{4}\right)$.

For the zero equilibrium point *P*_0_ = (0, 0, 0, 0), the characteristic polynomial equation is yielded as

(23) $$\begin{array}{l} P(\lambda )=\det(1\lambda -J)\\ \;=(\lambda +1)[\lambda^{3}+0.4\lambda^{2}+(7ka-3.52)\lambda +3.64ka-5.2]=0 \end{array}$$

which implies the eigenvalues of *P*_0_ is only related to the memristor inner parameter *a* but not associated with the memristor inner parameter *b*. When *a* = 1, it is confirmed by Routh-Hurwitz criteria that *P*_0_ is stable if *k* ≥ 1.4286, otherwise it is unstable if *k* < 1.4286. Taking *k* = 0.8 and *a* = 1 as an example, the eigenvalues of *P*_0_ is derived as λ_1_ = 0.7684, λ_2, 3_ = −0.5842 ± j1.6237, λ_4_ = −1, implying that the zero equilibrium point is an unstable saddle point.

For the two nonzero equilibrium points *P*_1_ = (δ_1_, δ_2_, δ_3_, δ_4_) and *P*_2_ = (η_1_, η_2_, η_3_, η_4_), however, the corresponding eigenvalues should be solved by using MATLAB numerical simulations. When *k* = 0.8 and *a* = 1, for different memristor inner parameter *b*, two nonzero equilibrium points *P*_1_ and *P*_2_, their corresponding eigenvalues, and the generating attractor types are shown in Table 2. Therefore, it can be seen that the nonzero equilibrium points *P*_1_ and *P*_2_ are asymmetric, *P*_1_ is a stable node-focus for *b* ≥ 0.44, and whereas *P*_2_ always is an unstable saddle-focus, leading to the emergence of coexisting asymmetric attractors' behaviors. Moreover, the results in Table 2 demonstrate that when the value of the parameter *b* increases, the dynamical behaviors of the hyperbolic-type memristor based HNN have transitions from unstable chaotic to unstable periodic and then to stable point behaviors, i.e., the dynamical behaviors of the HNN can be stabilized by the hyperbolic-type memristor.

**Table 2:** Non-zero equilibrium points, the corresponding eigenvalues, and the generating attractor types.

***b***	**Nonzero equilibrium points *P*_1_ and *P*_2_**	**Eigenvalues**	**Attractor type**
0	(1.7654, −0.3644, −0.5500, 0.9431) (−1.7654, 0.3641, 0.5500, −0.9431)	0.3660 ± j1.1781, −0.8889, −1.0000 0.3660 ± j1.1781, −0.8889, −1.0000	Double-scroll chaotic attractor
0.26	(2.1661, −0.5971, −0.6868, 0.9741) (−1.4755, 0.1974, 0.4529, −0.9006)	0.2290 ± j0.9261, −0.9751 ± j0.0484 0.4089 ± j1.3451, −0.7536, −1.0488	Right-chaotic spiral attractor and double-scroll chaotic attractor
0.41	(2.5629, −0.8138, −0.8270, 0.9882) (−1.3265, 0.1182, 0.4040, −0.8684)	0.0501 ± j0.6812, −0.9883 ± j0.0439 0.4041 ± j1.4327, −0.6655, −1.0749	Right-period-1 limit cycle and left-chaotic spiral attractor
0.43	(2.6399, −0.8541, −0.8555, 0.9899) (−1.3073, 0.1085, 0.3977, −0.8636)	0.0126 ± j0.6379, −0.9898 ± j0.0418 0.4022 ± j1.4447, −0.6533, −1.0784	Right-period-1 limit cycle and left-period-4 limit cycle
0.44	(2.6811, −0.8754, −0.8709, 0.9907) (−1.2978, 0.1037, 0.3946, −0.8612)	−0.0076 ± j0.6155, −0.9906 ± j0.0406 0.4010 ± j1.4508, −0.6472, −1.0802	Right-stable point attractor and left-period-2 limit cycle
0.46	(2.7737, −0.9226, −0.9062, 0.9922) (−1.2787, 0.0943, 0.3884, −0.8561)	−0.0536 ± j0.5675, −0.9920 ± j0.0380 0.3986 ± j1.4631, −0.6348, −1.0837	Right-stable point attractor and left-period-2 limit cycle
0.54	(3.2263, −1.1437, −1.0988, 0.9969) (−1.2031, 0.0588, 0.3642, −0.8346)	−0.2883 ± j0.3859, −0.9963 ± j0.0263 0.3855 ± j1.5148, −0.5847, −1.0981	Right-stable point attractor and left-period-3 limit cycle

Furthermore, it can be amusingly found from a larger number of numerical simulations that in the region of 0.44 ≤ *b* < 3.06, *P*_1_ is a stable node-focus and *P*_2_ is an unstable saddle-focus; but in the region of *b* ≥ 3.06, *P*_1_ disappears and the unique nonzero equilibrium point *P*_2_ maintains an unstable saddle-focus.

## Coexisting behaviors of asymmetric attractors

The initial conditions of four state variables are selected as (0, 0.1, 0, 0), (0, −0.1, 0, 0), and (1, 0, 0, 0), respectively, and the parameter *k* is set to 0.8, to 0.95, and to 1, respectively. When the memristor inner parameter *b* is adjusted in the region of [0, 0.6], the coexisting behaviors of asymmetric attractors in the hyperbolic-type memristor based HNN can be revealed by bifurcation diagrams, Lyapunov exponent spectra, and phase portraits. Note that Wolf's method reported in Wolf et al. (1985) is used to calculate the finite-time Lyapunov exponents, where MATLAB ODE45 algorithm with time-step 0.1 s and time-end 10 ks are utilized.

### Case 1 for *k* = 0.8

**Figure 5:**
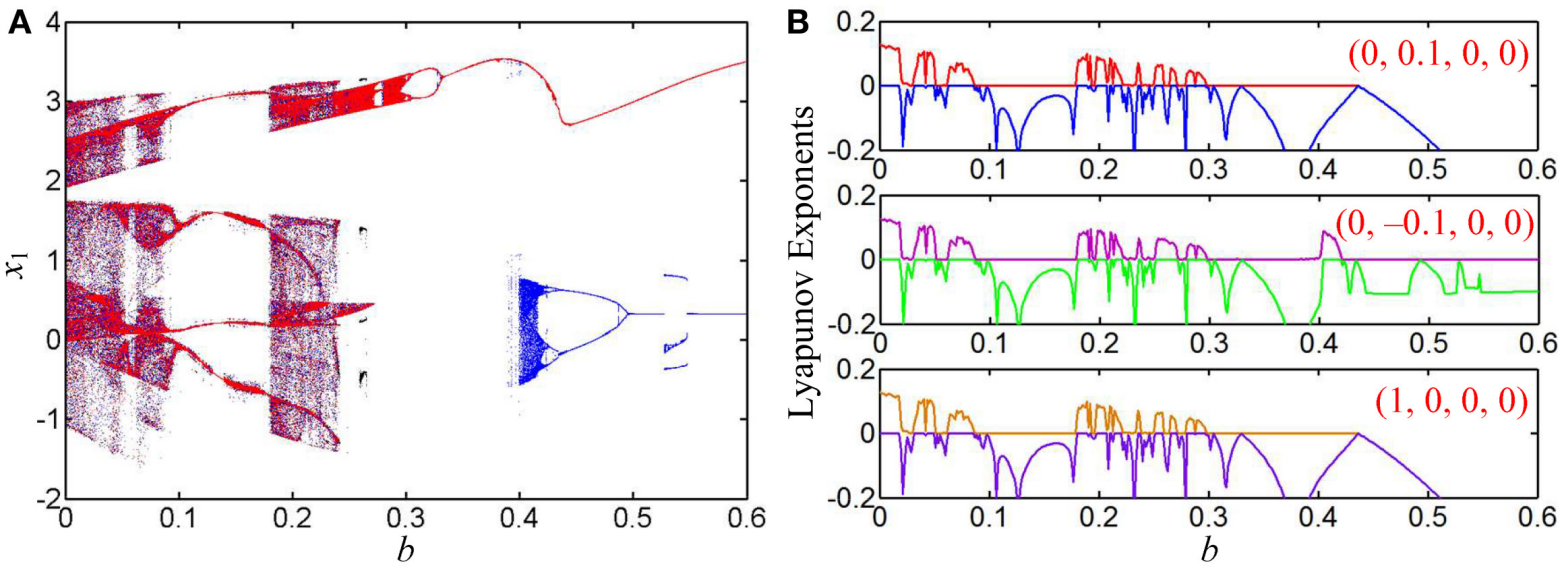
Coexisting behaviors of asymmetric attractors with the increase of *b*, where *k* = 0.8 and *a* = 1. **(A)** Bifurcation diagrams of the state variable *x*_1_, the red, blue, and black orbits corresponding to (0, 0.1, 0, 0), (0, −0.1, 0, 0), and (1, 0, 0, 0), respectively; **(B)** three kinds of first two Lyapunov exponents under different initial conditions.

When *k* = 0.8, the bifurcation diagrams of the state variable *x*_1_ and the corresponding first two Lyapunov exponents are shown in Figures 5A,B, respectively, from which unstable chaotic, unstable periodic, and stable point behaviors as well as period doubling bifurcation routes, tangent bifurcation routes, and crisis scenarios can be found. When the parameter *b* is gradually increased in the region of (0, 0.4), the dynamical behaviors of system Equation (8) are basically consistent for different initial conditions and their orbits all start from chaos, enter into a large periodic window with zero largest Lyapunov exponents via reverse period doubling bifurcation routes, then break into chaos via tangent bifurcation routes, further turn into chaos with narrow bands via crisis scenarios, and last degrade into period via reverse period doubling bifurcation routes.

**Figure 6:**
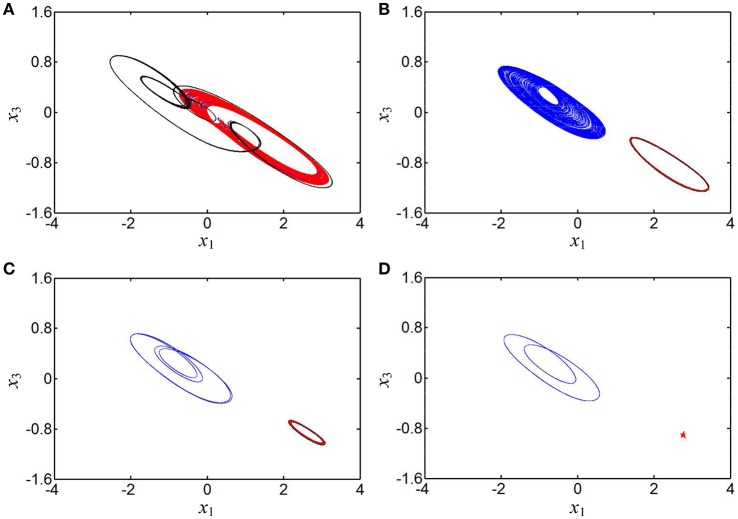
Phase portraits of asymmetrically coexisting attractors with different *b* in the *x*_1_ – *x*_3_ plane. **(A)** Coexistence of right-chaotic spiral attractor and double-scroll chaotic attractor at *b* = 0.26; **(B)** coexistence of right-period-1 limit cycle and left-chaotic spiral attractor at *b* = 0.41; **(C)** coexistence of right-period-1 limit cycle and left-period-4 limit cycle at *b* = 0.43; **(D)** coexistence of right-stable point attractor (marked with five-pointed star) and left-period-2 limit cycle at *b* = 0.46.

It is especially interesting that coexisting behaviors of asymmetric attractors can be also observed, which are mainly emerged in two regions of [0.2595, 0.2640] and [0.4, 0.6] due to the occurrences of the crisis scenarios under the initial conditions of (0, −0.1, 0, 0) and (1, 0, 0, 0). For some determined values of the parameter *b*, the phase portraits of coexisting asymmetric attractors in the *x*_1_ – *x*_3_ plane are depicted in Figure 6, where four different kinds of coexisting asymmetric attractors are exhibited. Based on the above theoretical analyses, it can be known that the generating chaotic attractors and limit cycles in Figure 6 are associated with two unstable saddle-foci and the convergent point attractor in Figure 6D is related to a stable node-focus. Consequently, the hyperbolic-type memristor based HNN is self-excited but not hidden (Danca and Kuznetsov, 2017).

### Case 2 for *k* = 0.95

**Figure 7:**
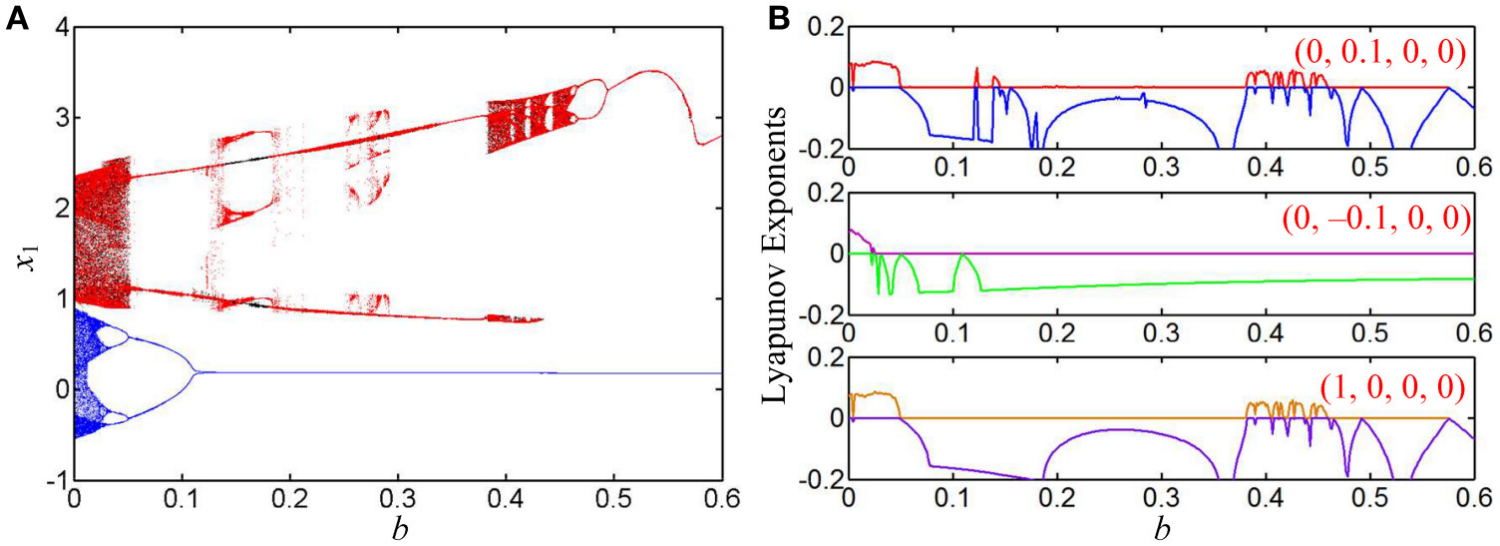
Coexisting behaviors of asymmetric attractors with the increase of *b*, where *k* = 0.95 and *a* = 1. **(A)** Bifurcation diagrams of the state variable *x*_1_, the red, blue, and black orbits corresponding to (0, 0.1, 0, 0), (0, −0.1, 0, 0), and (1, 0, 0, 0), respectively; **(B)** three kinds of first two Lyapunov exponents under different initial conditions.

When the coupling strength of the hyperbolic-type memristor is chosen as *k* = 0.95, the bifurcation diagrams of the state variable *x*_1_ and the corresponding first two Lyapunov exponents are shown in Figures 7A,B, respectively. Similarly, dynamical behaviors of chaotic attractors, limit cycles, point attractors, bifurcation routes, and crisis scenarios can be found in Figure 7. Differing from the dynamical behaviors in the case 1, the globally coexisting behaviors of asymmetric attractors are existed in this case.

**Figure 8:**
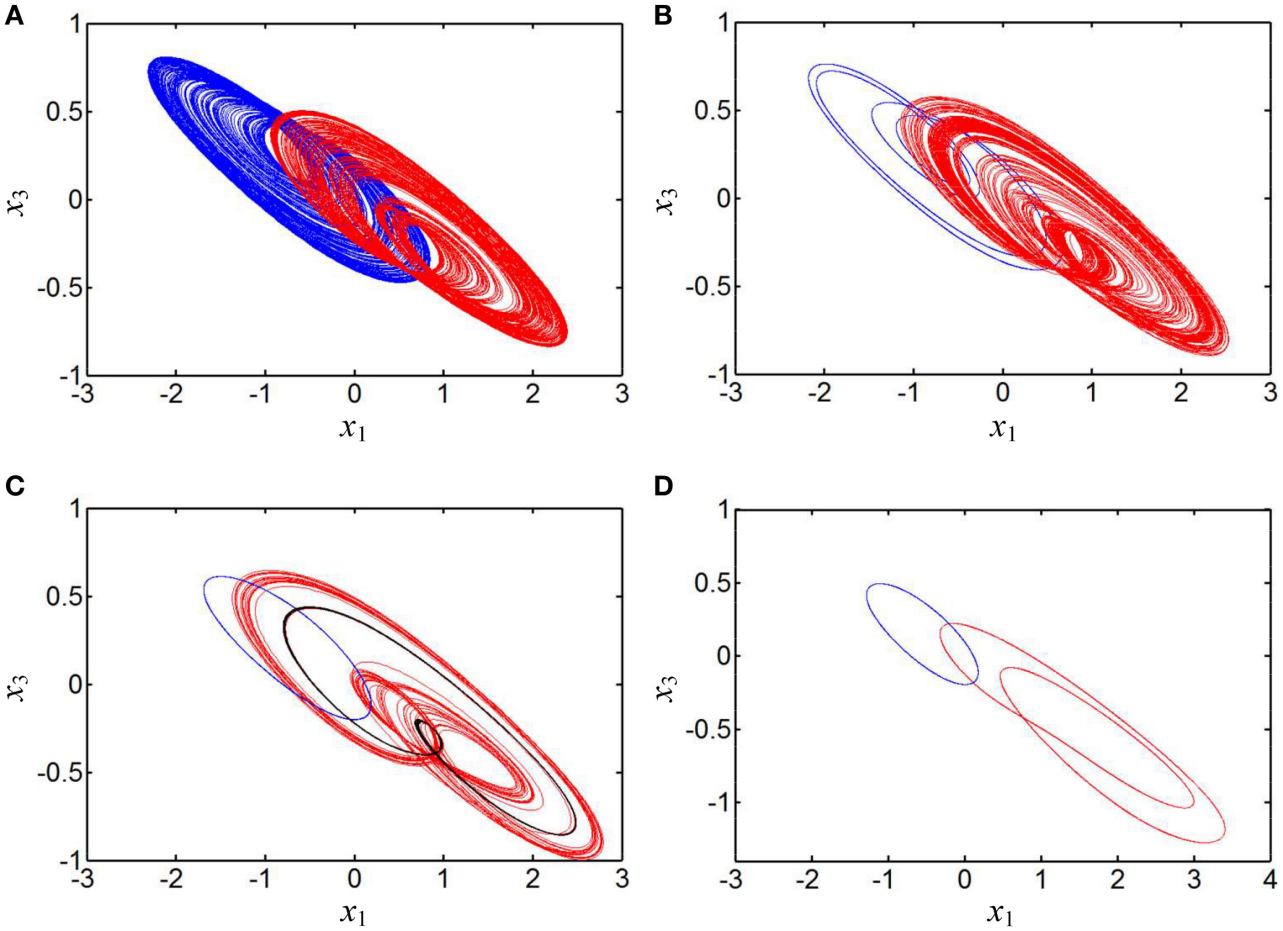
Phase portraits of asymmetrically coexisting attractors with different *b* in the *x*_1_ – *x*_3_ plane. **(A)** Coexistence of right- and left-chaotic spiral attractors at *b* = 0.01; **(B)** coexistence of right-chaotic spiral attractor and left-period-4 limit cycle at *b* = 0.04; **(C)** coexistence of right-chaotic spiral attractor, left-period-1 limit cycle, and right-period-2 limit cycle at *b* = 0.14; **(D)** coexistence of right-period-2 limit cycle and left-period-1 limit cycle at *b* = 0.46.

Several phase portraits of coexisting asymmetric attractors in the *x*_1_ – *x*_3_ plane are plotted in Figure 8. In special, the coexistence of three disconnected attractors at *b* = 0.14 are displayed in Figure 8C, where for the initial conditions (0, 0.1, 0, 0), a right-chaotic spiral attractor appears in the hyperbolic-type memristor based HNN and four Lyapunov exponents are *L*_1_ = 0.0321, *L*_2_ = 0, *L*_3_ = −0.4098, and *L*_4_ = −0.9715, respectively; whereas for the initial conditions (0, −0.1, 0, 0) and (1, 0, 0, 0), a left-period-1 limit cycle and a right-period-2 limit cycle are separately emerged in the system (8), the corresponding Lyapunov exponents are *L*_1_ = 0, *L*_2_ = −0.1190, *L*_3_ = −0.1193, and *L*_4_ = −1.0295, respectively, as well as *L*_1_ = 0, *L*_2_ = −0.1814, *L*_3_ = −0.1815, and *L*_4_ = −0.9698, respectively.

### Case 3 for *k* = 1

**Figure 9:**
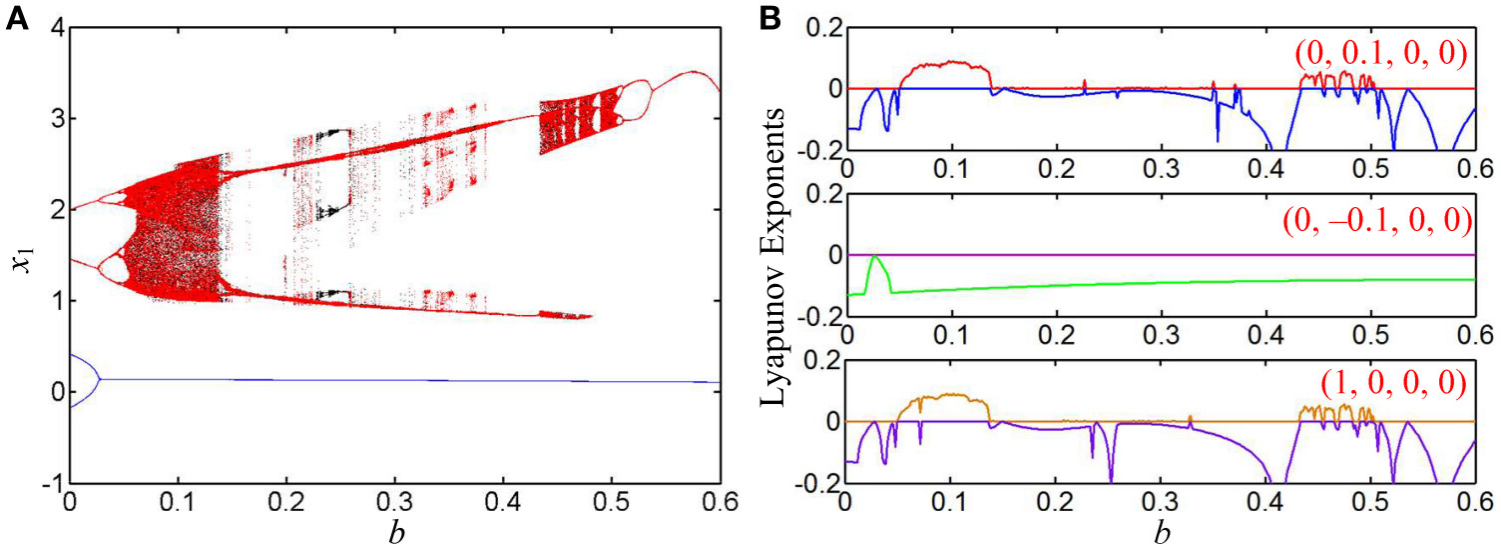
Coexisting behaviors of asymmetric attractors with the increase of *b*, where *k* = 1 and *a* = 1. **(A)** Bifurcation diagrams of the state variable *x*_1_, the red, blue, and black orbits corresponding to (0, 0.1, 0, 0), (0, −0.1, 0, 0), and (1, 0, 0, 0), respectively; **(B)** three kinds of first two Lyapunov exponents under different initial conditions.

When the coupling parameter is changed as *k* = 1, the bifurcation diagrams of the state variable *x*_1_ and the corresponding first two Lyapunov exponents are shown in Figures 9A,B, respectively. However, only for the initial conditions (0, 0.1, 0, 0) and (1, 0, 0, 0), complex dynamical behaviors including limit cycles with different periodicities, right-chaotic spiral attractors with different topologies, right-period-2 limit cycles with transient chaos, and stable point attractors as well as period doubling bifurcation routes, tangent bifurcation routes, and crisis scenarios can be discovered. Like as the dynamical behaviors in the case 2, the globally coexisting behaviors of asymmetric attractors can be also found in this case.

**Figure 10:**
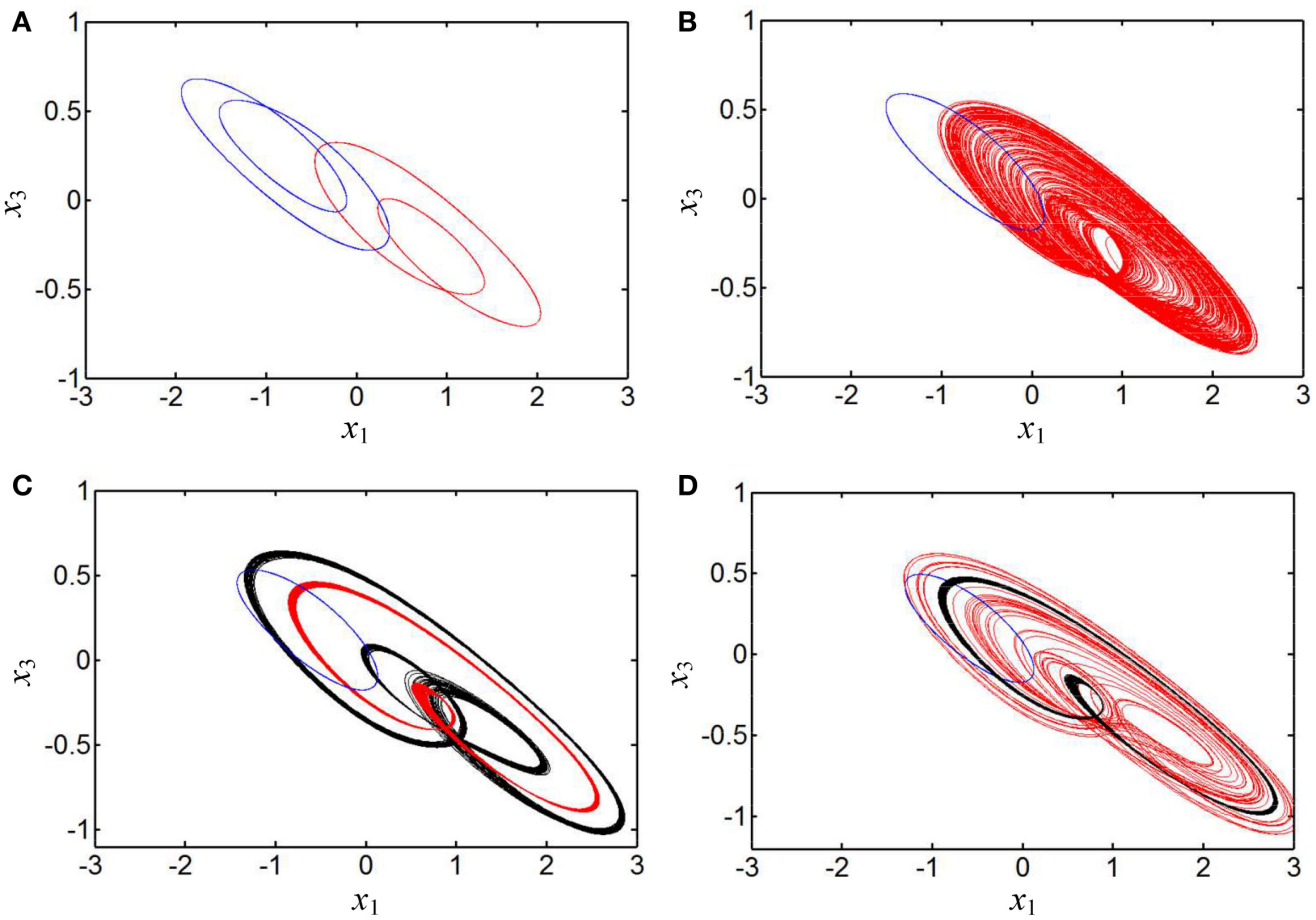
Phase portraits of asymmetrically coexisting attractors with different *b* in the *x*_1_ – *x*_3_ plane. **(A)** Coexistence of right- and left- period-2 limit cycles at *b* = 0.01; **(B)** coexistence of right-chaotic spiral attractor and left-period-1 limit cycle at *b* = 0.1; **(C)** coexistence of right-period-2 limit cycle, left-period-1 limit cycle, and right-period-3 limit cycle at *b* = 0.24; **(D)** coexistence of right-period-2 limit cycle with transient chaos, left-period-1 limit cycle, and right-period-2 limit cycle at *b* = 0.34.

Some phase portraits of coexisting asymmetric attractors in the *x*_1_ – *x*_3_ plane are demonstrated, as shown in Figure 10. It should be mentioned that two types of coexisting asymmetric multiple attractors' behaviors are generated, as shown in Figures 10C,D, in which a strikingly coexisting phenomenon of right-period-2 limit cycle with transient chaos, left-period-1 limit cycle, and right-period-2 limit cycle at *b* = 0.34 is presented in Figure 10D.

### Two remarks

But above all, different kinds of coexisting asymmetric attractors can be easily found in the hyperbolic-type memristor based HNN. It is significant to stress that the coexistence of two and three disconnected attractors revealed in the above three cases are all asymmetric, which are different from the symmetric coexistence of multiple attractors encountered in many nonlinear dynamical systems (Kengne et al., 2015; Bao et al., 2016a; Xu et al., 2016). Therefore, the hyperbolic-type memristor based HNN is a specific nonlinear dynamical system.

Additionally, according to the above three cases, it can be thereby concluded that the coupling strength and inner parameters of the hyperbolic-type memristor have significant effects on the dynamics of the hyperbolic-type memristor based HNN, i.e., the newly introduced hyperbolic-type memristor can cause the HNN initially in chaotic state to be stabilized to the stable state or can be used to generate chaotic signals in the HNN. In other words, the memristor can be regarded as a controller to realize the dynamical control of the HNN.

## PSPICE circuit simulations and hardware experiments

By using commercial discrete components, an experimental circuit can be made to verify the coexisting behaviors of asymmetric attractors in the hyperbolic-type memristor based HNN.

### Design and fabrication of hardware circuit

**Figure 11:**
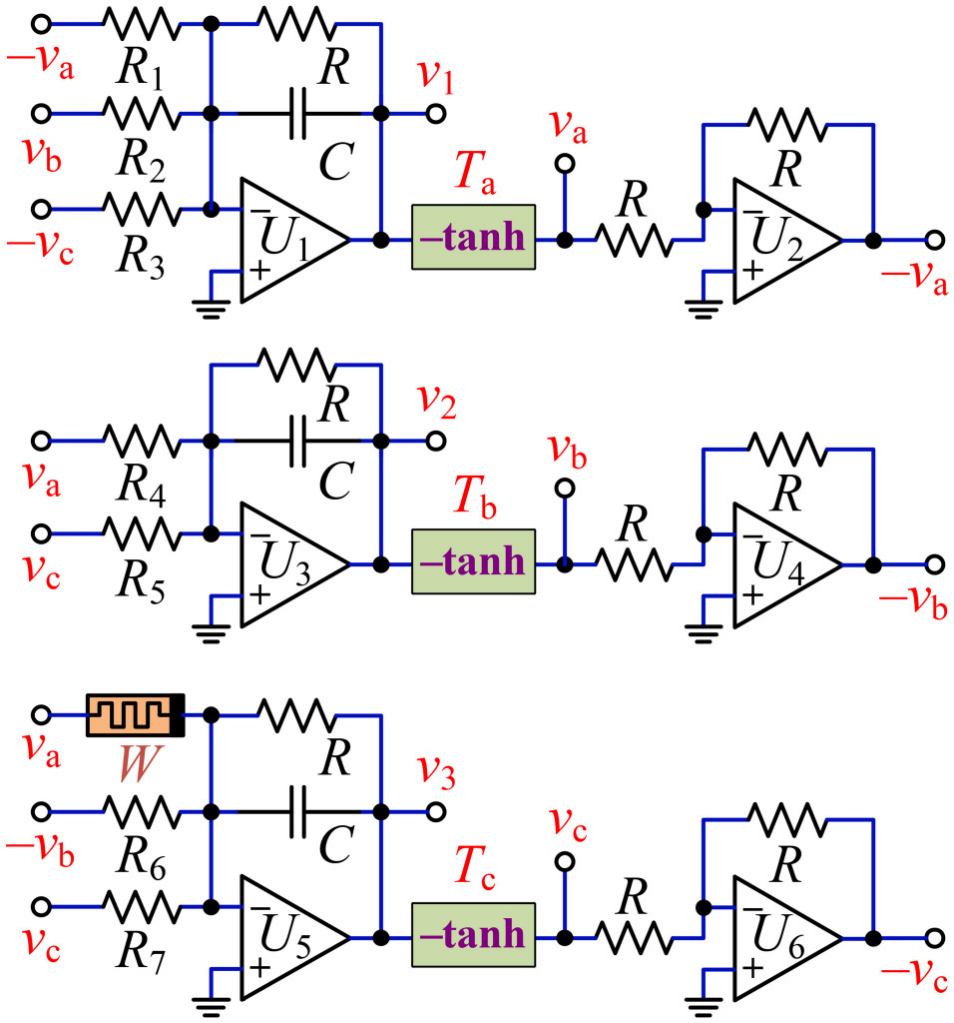
Circuit realization scheme of the hyperbolic-type memristor based HNN.

With the mathematical model of Equation (8), the hyperbolic-type memristor based HNN can be physically implemented by some operational amplifiers linked with resistors and/or capacitors (Bao et al., 2017), a nonlinear memristor, and three nonlinear function circuits. The circuit scheme for the memristive HNN is drawn in Figure 11, where the memristor *W* represents the proposed hyperbolic-type memristor emulator in Figure 1A and the three circuit modules *T*_a_, *T*_b_, and *T*_c_ marked by –tanh with solid box are the inverting hyperbolic tangent function circuit units drawn in Figure 1B.

The desired circuit in Figure 11 has four dynamic elements of three capacitors and a hyperbolic-type memristor *W*, corresponding to four state variables of *v*_1_, *v*_2_, *v*_3_, and *v*_0_, respectively. Therefore, the circuit state equations for Figure 11 are established as

(24) $$\begin{array}{l} RC\frac{\text{d}v_{1}}{\text{d}t}=-v_{1}-\frac{R}{R_{1}}\tanh(v_{1})+\frac{R}{R_{2}}\tanh(v_{2})-\frac{R}{R_{3}}\tanh(v_{3})\\ RC\frac{\text{d}v_{2}}{\text{d}t}=-v_{2}+\frac{R}{R_{4}}\tanh(v_{1})+\frac{R}{R_{5}}\tanh(v_{3})\\ RC\frac{\text{d}v_{3}}{\text{d}t}=-v_{3}+\left(\frac{R}{R_{a}}-\frac{gR}{R_{b}}\tanh(v_{0})\right)\tanh(v_{1})\\ \;-\;\frac{R}{R_{6}}\tanh(v_{2})+\frac{R}{R_{7}}\tanh(v_{3})\\ RC\frac{\text{d}v_{0}}{\text{d}t}=-v_{0}+\tanh(v_{1}) \end{array}$$

Assuming that the integrating time constant *RC* = 1 ms, the resistance and the capacitance can be chosen as *R* = 10 kΩ and *C* = 100 nF, respectively. Based on the element values of the weight matrix (7), other resistances are calculated as *R*_1_ = *R*/1.4 = 7.143 kΩ, *R*_2_ = *R*/1.2 = 8.333 kΩ, *R*_3_ = *R*/7 = 1.429 kΩ, *R*_4_ = *R*/1.1 = 9.091 kΩ, *R*_5_ = *R*/2.8 = 3.571 kΩ, *R*_6_ = *R*/2 = 5 kΩ, *R*_7_ = *R*/4 = 2.5 kΩ. It is significant to illuminate that two adjustable parameters of the coupling strength *k* and the inner parameter *b* are achieved by adjusting precision potentiometers *R*_*a*_ and *R*_*b*_ of the hyperbolic-type memristor emulator in Figure 1A, whose calculating relations between the resistances and the system parameters can be determined as *R*_*a*_ = *R*/*ka* kΩ and *R*_*b*_ = *gR*/*kb* kΩ, respectively.

**Figure 12:**
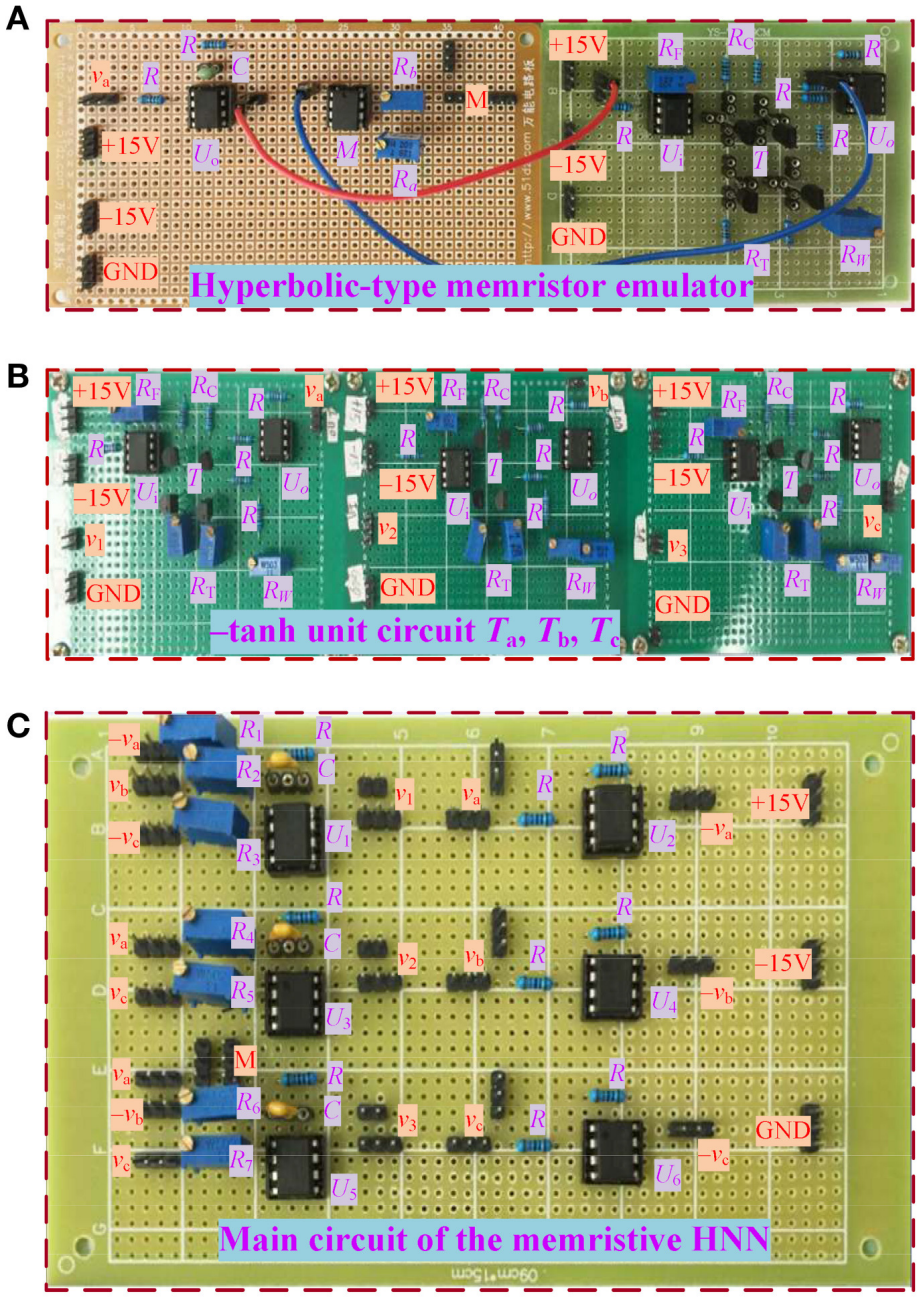
Hardware experimental breadboards using commercial discrete components. **(A)** Hyperbolic-type memristor emulator; **(B)** three inverting hyperbolic tangent function circuit units; **(C)** main circuit of the hyperbolic-type memristor based HNN.

With the circuit schemes displayed in Figures 1, 11, a circuit simulation model plotted by using PSPICE electronic circuit simulator and a hardware experimental circuit fabricated by commercial electronic components are gotten ready for validating the coexisting behaviors of asymmetric attractors in the hyperbolic-type memristor based HNN. The pictures of several circuit breadboards are photographed as depicted in Figure 12, where Figure 12A is the hyperbolic-type memristor emulator, Figure 12B is the three inverting hyperbolic tangent function circuit units, and Figure 12C is main circuit of the hyperbolic-type memristor based HNN. Additionally, the experimentally captured phase portraits are obtained by 4 channel digital oscilloscope.

### Simulated and captured results

**Figure 13:**
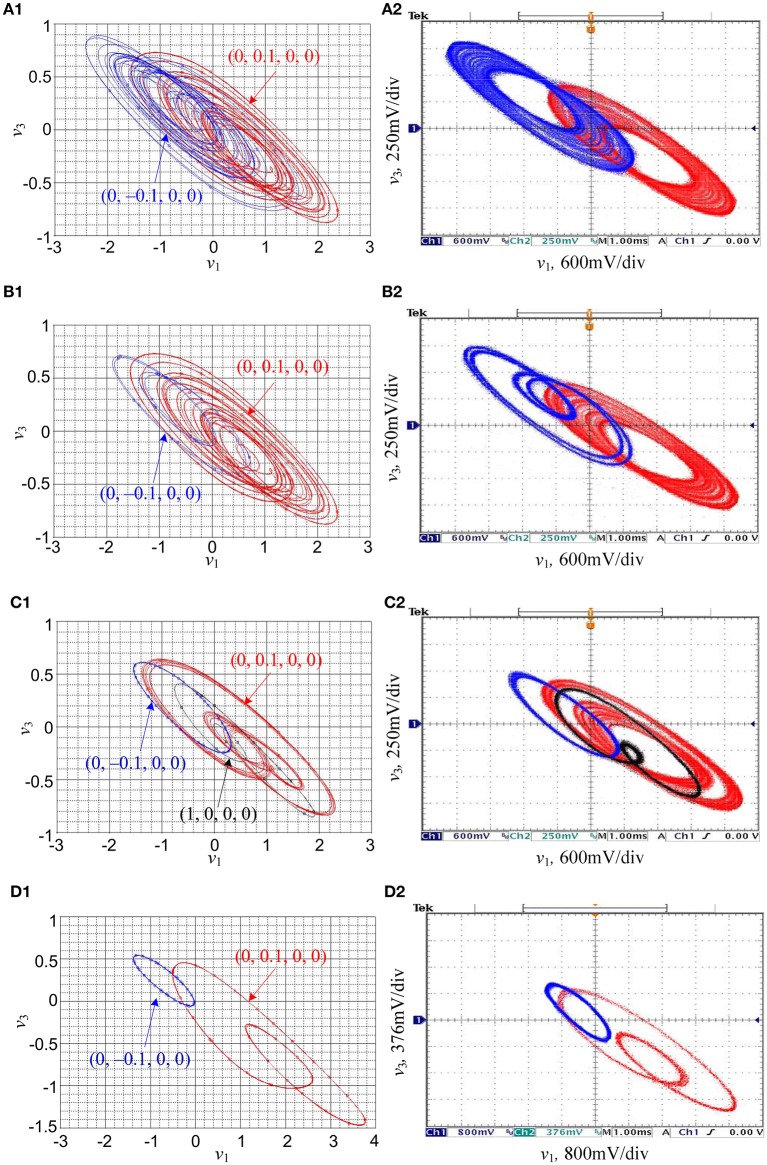
PSPICE simulated and experimentally captured phase portraits of coexisting asymmetric attractors in the *v*_1_–*v*_3_ plane, where **(A1,B1,C1,D1)** are PSPICE circuit simulations, and **(A2,B2,C2,D2)** are hardware experimental measurements. **(A)** Coexistence of right- and left-chaotic spiral attractors; **(B)** coexistence of right-chaotic spiral attractor and left-period-4 limit cycle; **(C)** coexistence of right-chaotic spiral attractor, left-period-1 limit cycle, and right-period-2 limit cycle; **(D)** coexistence of right-period-2 limit cycle and left-period-1 limit cycle.

Consider *k* = 0.95 as an example to perform PSPICE circuit simulations and hardware experiments of the memristive HNN. When *a* = 1, the resistance *R*_*a*_ is fixed as 10.53 kO and only the resistance *R*_*b*_ is adjustable. By adjusting the resistance *R*_*b*_ of the hyperbolic-type memristor emulator, some phase portraits in the *v*_1_ – *v*_3_ plane are simulated by PSPICE simulator with time step 10^−5^ and captured by a 4 channel digital oscilloscope in XY mode, as shown in Figure 13, where the orbits marked with red, blue, and black colors represent those triggered by three different initial values. It should be stressed that, in PSPICE circuit simulations the different initial values are assigned by setting different initial capacitor voltages, whereas during hardware circuit experiments the desired initial values are randomly achieved by the induced voltages of four capacitors through switching on and off the hardware circuit power supplies.

The simulated and captured results given in Figure 13 are consistent with numerical simulations given in Figure 8, which availably illustrate that the circuit realization form of the hyperbolic-type memristor based HNN can also display coexisting behaviors of asymmetric attractors. However, corresponding to Figures 13A–D, the values of the resistor *R*_*b*_ in PSPICE circuit simulations are 105.82 kΩ, 26.13 kΩ, 7.45 kΩ, and 2.21 kΩ, respectively, while the values of the resistor *R*_*b*_ in hardware circuit experiments are equal to 105.2 kΩ, 25.45 kΩ, 7.2 kΩ, and 2.13 kΩ, respectively. Due to the computational errors in PSPICE simulation (Kuznetsov et al., 2017) and the existence of parasitic parameters in the practical hardware circuit, the differences of the values of the resistor *R*_*b*_ indeed appear in PSPICE circuit simulations and hardware circuit experiments.

## Conclusion

In this paper, a new hyperbolic-type memristor emulator is presented and a novel hyperbolic-type memristor based 3-neuron HNN is thereby constructed. Based on the mathematical models and physical realization circuits, the frequency-dependent pinched hysteresis loops of the presented emulator are analyzed by numerical simulations and confirmed by hardware experiments, and coexisting behaviors of asymmetric attractors in the memristive HNN are revealed by numerical simulations and validated by PSPICE circuit simulations and hardware experiments. The research results demonstrate that the hyperbolic-type memristor has the stabilization effect on the chaotic HNN, which can cause the HNN initially in chaotic state to be stabilized to the stable state or can be used to generate chaotic signals in the HNN. Moreover, for different memristor inner parameters, different coexisting behaviors of asymmetric attractors are emerged under different initial conditions, implying the existences of multistable oscillation states in the memristive HNN. In particular, this fantastic phenomenon of the coexisting asymmetric attractors' behaviors has not yet reported in previous literature achievements.

## Author contributions

BB proposed the concept, designed the whole study and make final approval of the article. HQ performed experimental studies and prepared the manuscript. QX performed numerical simulations. MC edited the manuscript. JW constructed the experimental circuit. YY performed theoretical analyses.

### Conflict of interest statement

The authors declare that the research was conducted in the absence of any commercial or financial relationships that could be construed as a potential conflict of interest.
